# Effectiveness of Two Cold Water Immersion Protocols on Neuromuscular Function Recovery: A Tensiomyography Study

**DOI:** 10.3389/fphys.2018.00766

**Published:** 2018-06-26

**Authors:** Braulio Sánchez-Ureña, Daniel Rojas-Valverde, Randall Gutiérrez-Vargas

**Affiliations:** ^1^School of Human Movement Sciences and Quality of Life, Universidad Nacional, Heredia, Costa Rica; ^2^Exercise and Health Sciences Program, Universidad Nacional, Heredia, Costa Rica; ^3^Center for Research and Diagnosis in Health and Sport, Universidad Nacional, Heredia, Costa Rica

**Keywords:** recovery, tensiomyography, cold water immersion, muscle function, fatigue

## Abstract

Cold water immersion (CWI) has become a highly used recovery method in sports sciences, which seeks to minimize fatigue and accelerate recovery processes; however, tensiomyography (TMG) is a new method to analyze the muscle mechanical response as a recovery indicator after CWI protocols, this relative new tool of muscle function assessment, can lead to new information of understand fatigue recovery trough CWI. The objective of the study was to compare the effect of two CWI protocols, on neuromuscular function recovery. Thirty-nine healthy males (21.8 ± 2.8 years, 73.2 ± 8.2 kg, 176.6 ± 5.3 cm and body fat 13.5 ± 3.4%) were included in the study. Participants were grouped into a continuous immersion (12 min at 12 ± 0.4°C) group, intermittent immersion (2 min immersion at 12 ± 0.4°C + 1 min out of water 23 ± 0.5°C) group, and a control group (CG) (12 min sitting in a room at 23 ± 0.5°C). Afterward, the participants performed eight sets of 30 s counter movement jumps (CMJs) repetitions, with a 90 s standing recovery between sets. Muscle contraction time (Tc), delay time (Td), muscle radial displacement (Dm), muscle contraction velocity at 10% of DM (V10), and muscle contraction velocity at 90% of DM (V90) in rectus, biceps femoris, and CMJ were measured. Neither CWI protocol was effective in showing improved recovery at 24 and 48 h after training compared with the CG (*p* > 0.05), in any TMG indicator of recovery in either muscle biceps or rectus femoris, nor was the CMJ performance (*F*_(6,111)_ = 0.43, *p* = 0.85, ${\text{ω}}_{\text{p}}^{2}$ = 0). Neither CWI protocol contributed to recovery of the neuromuscular function indicator.

## Introduction

The recovery process is of particular importance to athletes who are required to perform optimally over subsequent training sessions and competitions (Rattray et al., 2015) Cold water immersion (CWI) has become well-known and the most frequently used recovery method by specialists in sports sciences, both among high performance athletes and amateur athletes, who seek to minimize fatigue and accelerate recovery processes (Calleja-González et al., 2016; Ihsan et al., 2016). In this way, several reviews of these methods (Burgess and Lambert, 2010; Versey et al., 2013; Ihsan et al., 2016) as well as meta-analyses (Leeder et al., 2012; Hohenauer et al., 2015; Sánchez-Ureña et al., 2015; Machado et al., 2016), have demonstrated the beneficial effect of these techniques on recovery, while other meta-analysis studies report no significant effect in recovery (Murray and Cardinale, 2015; Higgins et al., 2017). The literature reports that CWI includes the following physiological effects: decreased skin temperature and internal temperature (Peiffer et al., 2010), decreased acute inflammation, localized edema, and thigh muscle volume level (Vaile et al., 2008), decreased muscular pain sensation (Rowsell et al., 2011; Pointon et al., 2012; Delextrat et al., 2013; Minett et al., 2014; Sánchez-Ureña et al., 2017) and increased parasympathetic activity after exercise favoring recovery processes (Al Haddad et al., 2010; Stanley et al., 2012). It also improves the perception of recovery (Brophy-Williams et al., 2011; Stanley et al., 2012) and decreases the perception of fatigue (Rowsell et al., 2011; Delextrat et al., 2013). These studies used continuous and intermittent CWI protocols, but few studies have made a comparison between both protocols.

Neuromuscular function is the capacity to perform mechanical work as a result of muscular and nervous system function; because of the relevance of this process, the evaluation of muscle properties is fundamental to accessing the effectiveness of the recovery protocols (Halson, 2014). In the particular case of neuromuscular function and fatigue, Halson (2014) indicates that neuromuscular function can be assessed through a series of tests ranging from maximum voluntary contraction, to speed tests, countermovement jumps, or any other test that expresses aspects such as maximum force, flight time, contact time, speed of execution, and other contractile properties. Several studies have reported that CWI can accelerate the recovery of neuromuscular performance, expressed in the ability to repeat counter movement jumps (CMJs) (Vaile et al., 2008), improves performance in CMJ (Ascensao et al., 2011; Sánchez-Ureña et al., 2017), and benefits the recovery of isometric strength and muscular power (Vaile et al., 2008). Other studies indicate that CWI has no effect on neuromuscular function (De Nardi et al., 2011; Pournot et al., 2011).

As far as contractile capacities are concerned, tensiomyography (TMG) is a non-invasive method to access muscle contraction properties; a monophasic quadrangular electrical stimulation (0–110 mA) is applied to superficial muscles to assess it involuntary mechanical response, this technique provides information about muscle stiffness or muscle tone, muscle contraction time, and fatigue (Rey et al., 2012). TMG uses involuntary muscle response, contraction time, and muscle deformation as neuromuscular function indicators. TMG parameters are contraction time (Tc), indicating the muscle contraction velocity; muscle radial displacement (Dm); muscle belly radial stiffness (De Paula Simola et al., 2015); and muscle contraction velocity at 10 and 90% of Dm (V10 and V90). These represent the velocity of the muscle belly radial deformation (De Paula Simola et al., 2015). TMG parameters have been used as fatigue indicators, which correlate with gold standards of neuromuscular function as plyometric jumps, muscle force, creatine phosphokinase (CPK), and others (García-Manso et al., 2012; Hunter et al., 2012; De Paula Simola et al., 2015, 2016). For example, higher Tc indicates lower muscular speed of response due to fatigue (Hunter et al., 2012), and lower Dm represents and evaluates muscle stiffness; therefore a lower Dm indicates a high muscle tone and an excess of rigidity in muscle structures. This behavior is dependent on the sport and its characteristics (Tous-Fajardo et al., 2010).

According to the evidence, mechanical muscle alteration has been related to increased muscle stiffness, less activation of the muscle fibers, and exercise induced-muscle damage (EIMD) (García-Manso et al., 2012; Hunter et al., 2012; De Paula Simola et al., 2015). EIMD has provided an explanation for the muscle damage response following a series of eccentric contractions; these will cause various outcomes, such as prolonged loss of muscle strength and delayed-onset muscle soreness (DOMS) (Ferreira-Junior et al., 2014). The day-to-day reliability of TMG parameters has been investigated and reported as high, with values between ICC = 0.84 and ICC = 0.95 (Tous-Fajardo et al., 2010; Rey et al., 2012; Simunic, 2012; Ditroilo et al., 2013; De Paula Simola et al., 2015). Only one study has analyzed the effect of CWI on the tensiomyographic indicators (García-Manso et al., 2011). It reported that significant decreases in muscle radial displacement, but no significant differences in contraction time variable, and the acute effect of CWI on these indicators was discussed without using a pre-CWI fatigue protocol. Based on the evidence, this study’s aim was to compare the effectiveness of two CWI protocols on neuromuscular function of recovery at 24 and 48 h post-exercise.

## Materials and Methods

### Experimental Design

An experimental randomized 3 × 3 repeated measure was used. The effectiveness of two CWI protocols was tested immediately after an exhaustion fatigue protocol was performed. The participants were randomly divided into three groups (13 subjects per group) using a table of random numbers: an intermittent cold water immersion (ICWI) group, a continuous cold water immersion (CCWI) group, and a control group (CG).

### Participants

A total of 39 healthy males participated (21.8 ± 2.8 years, 73.2 ± 8.2 kg, 176.6 ± 5.3 cm and body fat 13.5 ± 3.4%). Inclusion criteria were as follows: male, active student, has a 20% or lower fatigue index in continuous 30 s CMJs, and no knee or ankle injuries in the 4 weeks prior to the tests. Participation in the study was voluntary, and the experimental procedures, associated risks, and benefits were explained to each player and documented in a signed informed consent form. The protocol was reviewed and approved by the ethics committee of the National University of Costa Rica, N° P-006-2015.

### Devices

#### Body Composition

A HD-313 Tanita (Tanita Corporation, Tokyo, Japan) was used to assess the total body mass (kg) with a precision of ±0.1 kg. Height was measured using a wall stadiometer. Fat percentage was calculated using the Jackson and Pollock formula on skinfold data from seven sites (chest, midaxillary region, subscapular region, triceps, suprailiac, abdomen, and thigh) (Pollock and Wilmore, 1990) using a Lange skinfold caliper from Beta Technology (Cambridge, United Kingdom). These measurements were taken under the International Society for the Advancement of Kinanthropometry protocol (Stewart et al., 2011), and all participants were measured by an experienced researcher.

#### Counter Movement Jump

This was measured by an Axon Jump (Bioingenieria Deportiva, San Martín, Argentina), with Smart Axon 4.02 software. The CMJ was executed following the Bosco protocol. The subjects were asked to stand on the platform with legs separated shoulder-width apart, and the hands on the waist. Given a signal, they made an explosive jump. This test has a test–retest reliability of ICC = 0.98 (Markovic et al., 2004).

#### Neuromuscular Properties

A tensiomyography (TMG) (TMG, Ljubljana, Slovenia) was used to assess muscle properties of the rectus femoris (RF) knee extensor, hip flexor muscle, and the long head of the biceps femoris (BF) knee flexor and hip extensor muscle from both lower limbs; the average value from both legs was used for further analyses as in other similar studies (De Paula Simola et al., 2015).

Participants were asked to remain relaxed. For RF the participants were in supine position and a cushioned pad was used to fix the knee at 120°; for the BF a prone position was required and a cushioned pad was used to fix the knee joint at 150°.

The participants were asked to remain in a rest position for 5 min. After cleaning the area, two 5 cm^2^ adhesive electrodes (TheraTrode^®^, TheraSigma, Orange, CA, United States) were placed on the respective muscles at a 5 cm distance from each other avoiding the tendon insertions; the negative electrode was placed distal from the measurement point (García-García et al., 2013). The measurement point was set at the maximal radial circumference of each muscle; it was established visually and by palpation of the muscle during a voluntary contraction. The electrodes were connected to an electrical stimulator (TMG-S2 doo, Ljubljana, Slovenia) that triggers a quadrangular, monophasic, 1 ms pulse duration wave between 0.1 and 110 mA. An accurate digital displacement transducer (GK 40, Panoptik doo, Ljubljana, Slovenia) was positioned perpendicular to the previously established measurement point of muscle belly (De Paula Simola et al., 2015).

The measurement protocol started triggering at a 40 mA electrical stimulus to induce a muscle contraction, whereby the electrical stimulus was increased by 20 mA until the maximal radial displacement was obtained; the electrical stimuli was then separated from each other by 10 s rest, to avoid fatigue or post-tetanic activation (De Paula Simola et al., 2016).

From TMG measurements the following parameter were obtained: muscle contraction time (Tc) expressed in ms (ICC = 0.92) (Tous-Fajardo et al., 2010; Benítez-Jiménez et al., 2013) [maximum radial muscle displacement (Dm) in mm (ICC = 0.94–0.97)] (Tous-Fajardo et al., 2010; Benítez-Jiménez et al., 2013); the muscle contraction speed from the onset of electrical stimulation until it reached 10% (V10) and 90% (V90) of Dm, expressed in mm/mm/s^-1^ was obtained by the formula developed by De Paula Simola et al. (2015, 2016) (ICC = 0.92–0.94; CV = 4.9–9.9%).

#### Rate of Perceived Exertion (RPE)

This variable was measured using a modified “Borg” 0–10 visual analogic scale. The RPE was measured immediately after the fatigue protocol.

### Procedure

The base line and post-measurements were made in the same hour and in the following order: height, body mass, body fat %, CMJ, and TMG. They were performed in a controlled laboratory at 23 ± 0.5°C.

#### Fatigue Protocol

Before the fatigue protocol was implemented, a 10 min × 4.1 Mph warm up was performed. After that, the participants undertook eight sets of as many CMJ repetitions as possible in 30 s; each set was separated by a 90 s stand rest.

#### Recovery Protocols

**FIGURE 1:**
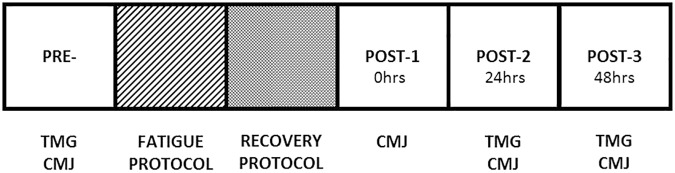
Schematic study design.

The immersions were conducted immediately after the fatigue protocol (RPE, Control Group = 9.7 ± 0.82; ICWI Group = 9.3 ± 1.4; CCWI = 9.4 ± 0.76, no significant differences, *F*_(2,39)_ = 0.57, *p* = 0.56) in a rounded 0.75 m deep and 3 m circumference pool where the subjects were sitting, with legs fully extended, and the water reaching navel height. The water was cooled by ice cubes. The temperature of the water was controlled minute by minute. The CWI and control protocols were performed as follows: CG, 12 min sitting in a 23 ± 0.5°C room; ICWI, 12 min intermittent immersions of 2 min inside (12 ± 0.4°C) and 1 min outside (23 ± 0.5°C); CCWI, 12 min continuous immersion at 12 ± 0.4°C. Immediately after the recovery protocol, a single CMJ was measured. The CMJ and TMG were measured 24 and 48 h post-CWI or control protocols (see **Figure 1**).

### Statistical Analysis

Descriptive statistics were employed using the mean (M) and standard deviations (± SD). Results are expressed as means ± standard deviation (SD). The normality of the data for each of the variables was checked by the Shapiro–Wilk test and the Levene test for homogeneity of variance; Box’s M test and Mauchly’s sphericity were used to describe the homogeneity of the covariance matrices of the dependent variables. Data of CMJ and TMG were subjected to a 2 (condition) × 2 (period) mixed model ANOVA with an a previously set alpha of *p* < 0.05. The *post hoc* analysis was undertaken by the Bonferroni method. The magnitudes of the differences for all variables were analysed using the partial omega squared (${\text{ω}}_{\text{p}}^{2}$) for ANOVA analysis. The ${\text{ω}}_{\text{p}}^{2}$ values were qualitatively interpreted using the following thresholds: ≤0.01 small, ≤0.06 medium, and ≤0.14 large (Cohen, 1988). The data analysis was performed using the Statistical Package for the Social Sciences (SPSS, IBM, SPSS Statistics, V 22.0, Chicago, IL, United States).

## Results

**FIGURE 2:**
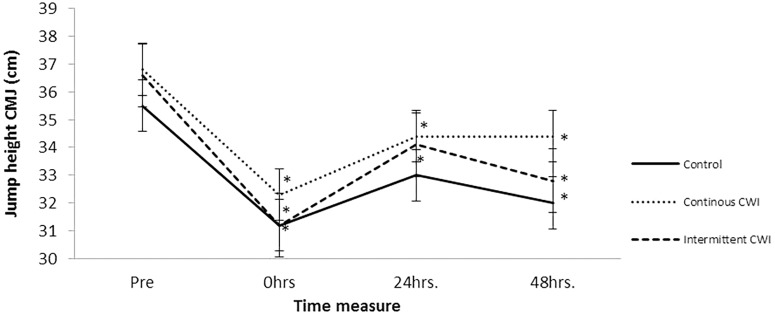
Comparison of counter movement jump behavior between groups by measure moment.

**Figure 2** shows the contrast between groups by measurement moment for the CMJ variable and the main effect into each group. The results showed no significant interaction between group and measurement moment (*F*_(6,111)_ = 0.43, *p* = 0.85, ${\text{ω}}_{\text{p}}^{2}$ = 0). After the main effects analysis, there was a significant difference between measurement moments (*F*_(3,35)_ = 14.13, *p* < 0.001, ${\text{ω}}_{\text{p}}^{2}$ = 0) as follows: pre-CG CMJ results were significantly -9.3% lower (31.2 ± 7 vs. 34.4 ± 5.8) than 0 h, -7% (32 ± 5.7 vs. 34.4 ± 5.8) and at the 48-h measurement point (*p* = 0.028, *p* = 0.030, respectively); the pre-continuous CWI group CMJ results were significantly -12.2% lower than 0 h (32.3 ± 6.7 vs. 36.8 ± 5), -6.5% lower (34.4 ± 6.2 vs. 36.8 ± 5) at 24 h and -6.5% lower (34.4 ± 5.9 vs. 36.8 ± 5) than 48-h measures (*p* = 0.004, *p* = 0.018, *p* = 0.033, respectively); the pre-intermittent CWI, CMJ results were significantly -14.8% lower at 0 h (31.2 ± 4 vs. 36.6 ± 5) (*p* = 0.001), -6.8% lower (34.1 ± 6 vs. 36.6 ± 5) at 24 h (*p* = 0.015), and -10.4% lower at 48 h (32.8 ± 6 vs. 36.6 ± 5) (*p* = 0.001). There was no main effect of group (*F*_(2,37)_ = 0.40, *p* < 0.67, ${\text{ω}}_{\text{p}}^{2}$ = 0).

Supplementary Table 1 shows the TMG rectus femoris intergroup contrasts by measurement moment. There was no TMG rectus femoris interaction between groups or measure moments for any of the TMG BF variables. These results suggest that the CWI protocol does not contribute to the TMG recovery of the rectus femoris as a neuromuscular function indicator.

Supplementary Table 2 shows the TMG biceps femoris inter groups contrast by measure moment. There was no TMG biceps femoris interaction between groups or measure moment for any of the TMG BF variables. These results suggested that the CWI protocol does not contribute to the TMG recovery of the biceps femoris as a neuromuscular function indicator.

## Discussion

The present study aimed to analyze the effects on recovery of two CWI protocols, including a continuous protocol (12 min of immersion at 12 ± 0.4°C) and an intermittent protocol (6 min × 2 min immersion at 12 ± 0.4°C + 1 min out of the water at room temperature) after exercise, compared to passive recovery. The results obtained in the present study indicate that immersions in cold water do not contribute significantly to the recovery of the muscular function evaluated by tensiomyographic indicators and functions such as CMJ jumping ability, regardless of the protocol used, no significant differences were found between groups on any of the variables analyzed.

The above findings were different from those reported by Sánchez-Ureña et al. (2017) (CCWI, 12 min at 12°C vs. ICWI, 4 min × 2 min: 1 min at 12°C) and Vaile et al. (2008) (CCWI, 14 min at 15°C), who identified significant differences between groups at 24 and 48 h after treatment in CMJ performance. However, these coincided with reports from most studies where this variable was analyzed, in which there was also no significant difference in CMJ jump height at post-immersion measurements 24 and 48 h later [De Nardi et al., 2011 (CCWI, 8 min at 15°C vs. ICWI, 2 min × 2 min: 2 min at 15°C) and Pournot et al., 2011 (CCWI, 15 min at 10°C)]. In particular, in the study carried out by Ascensao et al. (2011) (CWI, 10 min at 10°C), significant differences were reported between the CG and CWI group at 24 h, but in this case the values of the group of immersions were even smaller than the group of thermo-neutral immersions; that is, they reported a detrimental effect of the immersions in cold water. Recent meta-analytic studies report a low and negative effect of CWIs on the ability to jump at 24 h (ES = -0.30 [-0.96, 0.35]) (Murray and Cardinale, 2015). On the other hand (Higgins et al., 2017) also report that CWI did not show significant effect sizes in variables such as the CMJ jump at 24 h (*p* = 0.05, 95% CI -0.004 to 0.578).

To explain the behavior of the results obtained, Takeda et al. (2014) indicate that CMJ has usually been considered an indicator of neuromuscular performance, because after exercise-induced muscle damage, a decrease in CMJ is a result of impaired neuromuscular function and efficiency, due to the reduction of both the frequency and the intensity, by which the nerve impulse reaches the muscle. However, Pruscino et al. (2013) and Higgins et al. (2017) point out that the recovery of neuromuscular function depends not only on issues related to reducing the damage induced by exercise, but is also influenced by other physiological factors, such as muscle activation, muscle coordination, and level of fiber recruitment in the motor plate by the nervous system. Nevertheless, some studies (Rowsell et al., 2011; Higgins et al., 2013) indicate that recovery of EIMD is influenced by aspects such as the level of training and adaptive capacity.

On the other hand, a number of studies (Buchheit et al., 2011; Rowsell et al., 2011; Higgins et al., 2013) mention that the CMJ may not be sensitive enough to evaluate the recovery of neuromuscular function in trained athletes as these are influenced by psychological factors, such as a high level of motivation, competitiveness, exercise tolerance and pain tolerance, thereby allowing them to perform adequately when making maximum efforts in implementing a CMJ test.

To confirm the findings of this study, further trials are needed where these variables are analyzed; indicators of muscle function at the maximum voluntary contraction and torque, or biochemical indicators associated with muscle damage, such as CPK and lactate dehydrogenase (LDH), should be measured in addition to the subjective indicators of late-onset muscular pain (DOMS).

On the other hand, the implementation of recovery protocols performed in this study have no effect on mechanical fatigue indicators measured by the TMG, among which are Tc, Dm, V10, and V90. Dm behavior has been associated with an increase in muscle tone (García-Manso et al., 2012), and decrease in muscle fiber activation as a response to exercise induced fatigue (Hunter et al., 2012; De Paula Simola et al., 2015). In contrast, the present study shows a trend for a small decrease in the Dm behavior in the CG and García-Manso et al. (2011) reported significant differences in Dm during ICWIs (4 min × 4 min at 4°C) compared to a CG (no exercise was performed prior to the CWI/control interventions).

As for Tc (*p* = 0.89 in RF, *p* = 0.39 in BF) neither the study by De Paula Simola et al. (2015) nor the present study reported any differences over time after the exercise intervention. In contrast, De Paula Simola et al. (2015, 2016) reported that Dm, V10, and d V90 were able to detect fatigue after eccentric exercise. Despite this, in the present study, both CWI protocols were not effective in promoting superior recovery to the mechanical properties of the rectus femoris and the biceps femoris.

A possible explanation of why TMG indicators and the CMJ differ in the behavior of muscular fatigue at 24 and 48 h within the groups is that the TMG test was measured only in a muscle (rectus and biceps femoris) but the CMJ test included other muscles in the jump technique; the fatigue in these other muscles may explain the discrepancy between the TMG variables and CMJ. In future studies, it will be necessary to analyze other muscles, such as the gluteal and vastus femoris for example.

## Conclusion

Both CWI, continuous and intermittent protocols, were ineffective for promoting superior recovery of CMJ performance and TMG muscle mechanical responses.

## Practical Applications

A single CWI recovery protocol, regardless of whether it is intermittent or continuous for 12 min, is not capable of recovering the functional and mechanical muscle properties of rectus femoris nor biceps femoris after a strenuous eccentric exercise in active men. It is necessary to explore the role of frequency and protocols (time immersion and water temperature) of CWI application during the hours and days after strenuous exercise, and it would be of much practical interest to inquire about the effects of different recovery techniques on the mechanical functions of the muscle.

## Author Contributions

BS-U: designed the research study, conducted the experiments, acquired and analyzed the data, and wrote the final version of the manuscript. DR-V: designed the research study, acquired and analyzed the data, and wrote the final version of the manuscript. RG-V: designed the research study and acquired and analyzed the data.

## Conflict of Interest Statement

The authors declare that the research was conducted in the absence of any commercial or financial relationships that could be construed as a potential conflict of interest.

## References

[B1] Al Haddad H. Laursen P. Chollet D. Lemaitre F. Ahnire S. Buchheit M. (2010). Effect of cold or thermo neutral water immersion on post-exercise heart rate recovery and heart rate variability indices. *Auton. Neurosci.* 156 111–116. 10.1016/j.autneu.2010.03.017 20403733

[B2] Ascensao A. Leite M. Rebelo A. N. Magalhäes S. Magalhäes J. (2011). Effects of cold water immersion on the recovery of physical performance and muscle damage following a one-off soccer match. *J. Sports Sci.* 29 217–225. 10.1080/02640414.2010.526132 21170794

[B3] Benítez-Jiménez A. Fernández-Roldán K. Montero-Doblas J. M. Romacho-Castro J. A. (2013). Fiabilidad de la tensiomiografía (TMG) como herramienta de valoración muscular [Reliability of tensiomiography (TMG) as a muscle assessment tool]. *Rev. Intern. Med. Cien. Act. F ísica Deporte* 13 647–656.

[B4] Brophy-Williams N. Landers G. Wallman K. (2011). Effect of immediate and delayed cold water immersion after a high intensity exercise session on subsequent run performance. *J. Sports Sci. Med.* 10 665–670. 10.1016/j.jsams.2011.11.238 24149556 PMC3761518

[B5] Buchheit M. Horobeanu C. Mendez-Villanueva A. Simpson B. M. Bourdon P. C. (2011). Effects of age and spa treatment on match running performance over two consecutive games in highly trained young soccer players. *J. Sports Sci.* 29 591–598. 10.1080/02640414.2010.546424 21337251

[B6] Burgess T. Lambert M. (2010). The efficacy of cryotherapy on recovery following exercise-induced muscle damage. *Inter. Sport Med. J.* 11 258–277.

[B7] Calleja-González J. Terrados N. Mielgo-Ayuso J. Delextrat A. Jukic I. Vaquera A. (2016). Evidence based post-exercise recovery strategies in basketball. *Phys. Sports Med.* 44 74–78. 10.1080/00913847.2016.1102033 26512912

[B8] Cohen J. (1988). *Statistical Power Analysis for the Behavioral Sciences.* New York, NY: Routledge Academic.

[B9] De Nardi M. La Torre A. Barassi A. Ricci C. Banfi G. (2011). Effects of cold-water immersion and contrast-water therapy after training in young soccer players. *J. Sports Med. Phys. Fitness* 51 609–615. 22212263

[B10] De Paula Simola R. Á. Raeder C. Wiewelhove T. Kellmann M. Meyerm T. M. Pfeiffer M. (2016). Muscle mechanical properties of strength and endurance athletes and changes after one week of intensive training. *J. Electromyogr. Kinesiol.* 30 73–80. 10.1016/j.jelekin.2016.05.005 27317976

[B11] De Paula Simola R. Á Harms N. Raeder C. Kellmann M. Meyer T. Pfeiffer M. (2015). Assessment of neuromuscular function after different strength training protocols using tensiomyography. *J. Strength Cond. Res.* 29 1339–1348. 10.1519/JSC.0000000000000768 25474337

[B12] Delextrat A. Calleja-González J. Hippocrate A. Clarke N. D. (2013). Effects of sports massage and intermittent cold-water immersion on recovery from matches by basketball players. *J. Sports Sci.* 31 11–19. 10.1080/02640414.2012.719241 22935028

[B13] Ditroilo M. Smith I. J. Fairweather M. M. Hunter A. M. (2013). Long-term stability of tensiomyography measured under different muscle conditions. *J. Electromyogr. Kinesiol.* 23 558–563. 10.1016/j.jelekin.2013.01.014 23461833

[B14] Ferreira-Junior J. B. Bottaro M. Loenneke J. P. Vieira A. Vieira C. A. Bemben M. G. (2014). Could whole-body cryotherapy (below -100°C) improve muscle recovery from muscle damage? *Front. Physiol.* 5:247 10.3389/fphys.2014.00247 PMC4078193 25071592

[B15] García-García O. Cancela-Carral J. M. Martínez-Trigo R. Serrano-Gómez V. (2013). Differences in the contractile properties of the knee extensor and flexor muscles in professional road cyclists during the season. *J. Strength Cond. Res.* 27 2760–2767. 10.1519/JSC.0b013e31828155cd 23302746

[B16] García-Manso J. M. Rodríguez-Matoso D. Rodríguez-Ruiz D. Sarmiento S. de Saa Y. Calderón J. (2011). Effect of cold-water immersion on skeletal muscle contractile properties in soccer players. *Am. J. Phys. Med. Rehabil.* 90:356–363. 10.1097/PHM.0b013e31820ff352 21765254

[B17] García-Manso J. M. Rodríguez-Matoso D. Sarmiento S. de Saá Y. Vaamonde D. Rodríguez-Ruiz D. (2012). Effect of high-load and high-volume resistance exercise on the tensiomyographic twitch response of biceps brachii. *J. Electromyogr. Kinesiol.* 22 612–619. 10.1016/j.jelekin.2012.01.005 22341590

[B18] Halson S. (2014). Monitoring fatigue and recovery. *Sports Sci. Exch.* 27 1–6.

[B19] Higgins T. R. Climstein M. Cameron M. (2013). Evaluation of hydrotherapy, using passive tests and power tests, for recovery across a cyclic week of competitive rugby union. *J. Strength Cond. Res.* 27 954–965. 10.1519/JSC.0b013e318260ed9b 22796996

[B20] Higgins T. R. Greene D. A. Baker M. K. (2017). The effects of cold water immersion and contrast water therapy for recovery from team sport: a systematic review and meta-analysis. *J. Strength Cond. Res.* 31 1443–1460. 10.1519/JSC.0000000000001559 27398915

[B21] Hohenauer E. Taeymans J. Baeyens J. P. Clarys P. Clijsen R. (2015). The effect of post-exercise cryotherapy on recovery characteristics: a systematic review and meta-analysis. *PLoS One* 10:e0139028. 10.1371/journal.pone.0139028 26413718 PMC4586380

[B22] Hunter A. M. Galloway S. D. Smith I. J. Tallent J. Ditroilo M. Fairweather M. M. (2012). Assessment of eccentric exercise-induced muscle damage of the elbow flexors by tensiomyography. *J. Electromyogr. Kinesiol.* 22 334–341. 10.1016/j.jelekin.2012.01.009 22336641

[B23] Ihsan M. Waston G. Abbiss C. R. (2016). What are the physiological mechanisms for post-exercise cold water immersion in the recovery from prolonged endurance and intermittent exercise? *Sports Med.* 46 1095–1109. 10.1007/s40279-016-0483-3 26888646

[B24] Leeder J. Gissane C. Van Someren K. Gregson W. Howatson G. (2012). Cold water immersion and recovery from strenuous exercise: a meta-analysis. *Br. J. Sports Med* 46 233–240. 10.1136/bjsports-2011-090061 21947816

[B25] Machado A. F. Ferreira P. H. Micheletti J. K. de Almeida A. C. Lemes ÍR. Vanderlei F. M. (2016). Can water temperature and immersion time influence the effect of cold water immersion on muscle soreness? A systematic review and meta-analysis. *Sports Med.* 46 503–514. 10.1007/s40279-015-0431-7 26581833 PMC4802003

[B26] Markovic G. Dizdar D. Jukic I. Cardinale M. (2004). Reliability and factorial validity of squat and countermovement jump tests. *J. Strength Cond. Res.* 18 551–555. 10.1519/00124278-200408000-00028 15320660

[B27] Minett G. M. Duffield R. Billaut F. Cannon J. Portus M. R. Marino F. E. (2014). Cold-water immersion decreases cerebral oxygenation but improves recovery after intermittent-sprint exercise in the heat. *Scand. J. Med. Sci. Sports* 24 656–666. 10.1111/sms.12060 23458430

[B28] Murray A. Cardinale M. (2015). Cold applications for recovery in adolescent athletes: a systematic review and meta-analysis. *Extrem. Physiol. Med.* 4:17. 10.1186/s13728-015-0035-8 26464795 PMC4603811

[B29] Peiffer J. Abbiss C. Watson G. Nosaka K. Laursen P. B. (2010). Effect of cold water immersion on repeated 1-km cycling performance in the heat. *J. Sci. Med. Sport* 13 112–116. 10.1016/j.jsams.2008.08.003 18948061

[B30] Pointon M. Duffield R. Cannon J. Marino F. E. (2012). Cold water immersion recovery following intermittent-sprint exercise in the heat. *Eur. J. Appl. Physiol.* 112 2483–2494. 10.1007/s00421-011-2218-3 22057508

[B31] Pollock M. L. Wilmore J. H. (1990). *Exercise in Health and Disease*, 2nd Edn. Philadelphia, PA: W.B. Saunders.

[B32] Pournot H. Bieuzen F. DuYeld R. Lepretre P. Cozzolino C. Hausswirth C. (2011). Short term effects of various water immersions on recovery from exhaustive intermittent exercise. *Eur. J. Appl. Physiol.* 111 1287–1295. 10.1007/s00421-010-1754-6 21132438

[B33] Pruscino C. L. Halson S. Hargreaves M. (2013). Effects of compression garments on recovery following intermittent exercise. *Eur. J. Appl. Physiol.* 113 1585–1596. 10.1007/s00421-012-2576-5 23314683

[B34] Rattray B. Argus C. Martin K. Northey J. Driller M. (2015). Is it time to turn our attention toward central mechanisms for post-exertional recovery strategies and performance? *Front. Physiol.* 6:79. 10.3389/fphys.2015.00079 25852568 PMC4362407

[B35] Rey E. Lago-Peñas C. Lago-Ballesteros J. (2012). Tensiomyography of selected lower-limb muscles in professional soccer players. *J. Electromyogr. Kinesiol.* 22 866–872. 10.1016/j.jelekin.2012.06.003 22776612

[B36] Rowsell G. Coutts A. Raeburn P. Hill-Haas S. (2011). Effect of post-match cold water immersion on subsequent match running performance in junior soccer players during tournament play. *J. Sports Sci.* 29 1–6. 10.1080/02640414.2010.512640 21077001

[B37] Sánchez-Ureña B. Barrantes-Brais K. Ureña-Bonilla P. Calleja-González J. Ostojic S. (2015). Effect of water immersion on recovery from fatigue: a meta-analysis. *Eur. J. Hum. Mov.* 34 1–14.

[B38] Sánchez-Ureña B. Martínez-Guardado I. Crespo C. Timon R. Calleja-González J. Ibañez S. J. (2017). The use of continuous vs. intermittent cold water immersion as a recovery method in basketball players after training: a randomized controlled trial. *Phys. Sports Med.* 45 134–139. 10.1080/00913847.2017.1292832 28276987

[B39] Simunic B. (2012). Between-day reliability of a method for non-invasive estimation of muscle composition. *J. Electromyogr. Kinesiol* 22 527–530. 10.1016/j.jelekin.2012.04.003 22546361

[B40] Stanley J. Buchheit M. Peake J. (2012). The effect of post-exercise hydrotherapy on subsequent exercise performance and heart rate variability. *Eur. J. Appl. Physiol.* 112 951–961. 10.1007/s00421-011-2052-7 21710292

[B41] Stewart A. Marfell-Jones M. Olds T. Hans De Rideer J. (2011). *International Standards for Anthropometric Assessment.* Lower Hutt: ISAK

[B42] Takeda M. Sato T. Hasegawa T. Shintaku H. Kato H. Yamaguchi Y. (2014). The effects of cold water immersion after rugby training on muscle power and biochemical markers. *J. Sport Sci. Med.* 13 616–623. 25177190 PMC4126300

[B43] Tous-Fajardo J. Moras G. Rodriguez-Jimenez S. Usach R. Doutres D. M. Maffiuletti N. A. (2010). Inter-rater reliability of muscle contractile property measurements using non-invasive tensiomyography. *J. Electromyogr. Kinesiol* 20 761–766. 10.1016/j.jelekin.2010.02.008 20236839

[B44] Vaile J. Halson S. Gill N. Dawson B. (2008). Effect of hydrotherapy on the signs and symptoms of delayed onset muscle soreness. *Eur. J. Appl. Phys.* 102 447–455. 10.1007/s00421-007-0605-6 17978833

[B45] Versey N. Halson S. Dawson B. (2013). Water immersion recovery for athletes: effect on exercise performance and practical recommendations. *J. Sports Med.* 43 1101–1030. 10.1007/s40279-013-0063-8 23743793

