# Supplementary material for: Effectiveness of Two Cold Water Immersion Protocols on Neuromuscular Function Recovery: A Tensiomyography Study
Source: Front Physiol. 2018 Jun 26;9:766. doi: 10.3389/fphys.2018.00766 (PMC6028616; doi:10.3389/fphys.2018.00766)
Supplement: Supplementary file 1 [file Table_1.DOCX]

Table 1

*Comparison of TMG rectus femoris variables behavior between groups by measure moment.*

| Variable | Control Group | | | Continuous CWI | | | Intermittent CWI | | | *F* interaction | *p*  value | *ES*  *(*ω*_p_^2^)* |
| --- | --- | --- | --- | --- | --- | --- | --- | --- | --- | --- | --- | --- |
|  | Pre | 24h | 48h | Pre | 24h | 48h | Pre | 24h | 48h |  |  |  |
| TC (ms) | 28.3 ±4.3 | 28.9 ± 5.8 | 29.2 ± 6.3 | 29.1 ± 4.3 | 30.6 ± 3.6 | 30.6 ± 3.1 | 28.2 ± 3.6 | 29.1 ± 3.5 | 29.24 ± 4.53 | 0.27 | 0.89 | 0 |
| TD (ms) | 23 ± 1.3 | 23 ± 2 | 23.9 ± 2.3 | 24.4 ± 1.8 | 24 ± 1.7 | 24.4± 1.7 | 23.5 ± 2.1 | 24.7 ± 1.5 | 24.61 ± 1.3 | 0.57 | 0.68 | 0 |
| DM (mm) | 6.9 ± 1.8 | 6 ± 1.8 | 5.9 ± 1.7 | 7.8 ± 2.6 | 7.4 ± 2.1 | 7.9 ± 2.3 | 7.2 ± 2.9 | 6.6 ± 2.7 | 6.71 ± 2.54 | 1.11 | 0.35 | 0.003 |
| V10 (mm/s^-1^) | 29.9 ± 7.2 | 26.6 ± 7.7 | 25.7 ± 7.8 | 32.7 ± 10.9 | 30.4 ± 8.9 | 32.4 ± 9.6 | 30 ± 10.6 | 26.9 ± 10.5 | 27.26 ± 9.5 | 1.12 | 0.35 | 0.003 |
| V90 (mm/s^-1^) | 120.9 ± 29.0 | 106.6 ± 34.8 | 104.1 ± 31.8 | 133.2 ± 44.1 | 121.6 ± 34.1 | 130 ± 38.1 | 123 ± 44 | 109.9 ± 43.3 | 112 ± 40.3 | 0.88 | 0.45 | 0.003 |

Note: Tc = time of contraction, Td = delay time, Dm = Muscle Stiffness, Vrn = Normalize speed reaction, V10 = muscle contraction velocity of Dm, V90 = muscle contraction velocity 90% of Dm
