# Supplementary material for: Effectiveness of Two Cold Water Immersion Protocols on Neuromuscular Function Recovery: A Tensiomyography Study
Source: Front Physiol. 2018 Jun 26;9:766. doi: 10.3389/fphys.2018.00766 (PMC6028616; doi:10.3389/fphys.2018.00766)
Supplement: Supplementary file 2 [file Table_2.docx]

*Table 2*

*Comparison of TMG biceps femoris variables behavior between groups by measure moment.*

| Variable | Control grup | | | Continuos CWI | | | Intermittent CWI | | | *F* interaction | *p*  value | *ES*  *(*ω*_p_^2^)* |
| --- | --- | --- | --- | --- | --- | --- | --- | --- | --- | --- | --- | --- |
|  | Pre | 24h | 48h | Pre | 24h | 48h | Pre | 24h | 48h |  |  |  |
| Tc (ms) | 27.8 ± 4.3 | 29.6 ± 6.8 | 27.8 ± 7.8 | 27.4 ± 6.3 | 27.8 ± 6.1 | 26.3 ± 5.2 | 29.5 ± 6.6 | 28.3 ± 6.8 | 29.8 ± 8.1 | 1.03 | 0.39 | 0.001 |
| Td (ms) | 24.02 ± 3.+7 | 23.2 ± 2.5 | 23.9 ± 3.8 | 22.7 ± 2.1 | 23.2 ± 2 | 22.3 ± 1.8 | 23.7 ± 2.5 | 23.6 ± 2 | 23.5 ± 2.2 | 1 | 0.41 | 0.001 |
| Dm (mm) | 3.01 ± 1.2 | 2.9 ± 1.3 | 2.4 ± 1.4 | 4.1 ± 1.6 | 4.2 ± 1.6 | 3.9 ± 1.6 | 4.1 ± 2 | 4.2 ± 2.5 | 4.5 ± 2.4 | 1.14 | 0.34 | 0.003 |
| V10 (mm/s^-1^) | 13.24 ± 6.2 | 12.8 ± 6 | 11 ± 6.9 | 18.4 ± 5.6 | 18.4 ± 7 | 17.5 ± 7 | 17 ± 7.9 | 17.2 ± 9 | 18.5 ± 8.6 | 1.032 | 0.39 | 0 |
| V90 (mm/s^-1^) | 55.4 ± 28.7 | 52 ± 26.3 | 44.4 ± 28.4 | 76.6 ± 26.3 | 76.4 ± 28.1 | 72.5 ± 29.1 | 67.4 ± 29.5 | 68.8 ± 30.7 | 72.2 ± 27.2 | 1.34 | 0.26 | 0.009 |

Note: Tc = time of contraction; Td = delay time; Dm = muscle stiffness; Vrn = normalize speed reaction; V10 = muscle contraction velocity of DM; V90 = muscle contraction velocity 90% of DM
